# Processing Analysis of Nanoparticle Filled PTFE: Restrictions and Limitations of High Temperature Production

**DOI:** 10.3390/polym12092044

**Published:** 2020-09-08

**Authors:** Levente Ferenc Tóth, Patrick De Baets, Gábor Szebényi

**Affiliations:** 1 Soete Laboratory, Department of Electromechanical, Systems and Metal Engineering, Ghent University, Technologiepark Zwijnaarde 46, B-9052 Zwijnaarde, Belgium; levente.toth@ugent.be (L.F.T.); patrick.debaets@ugent.be (P.D.B.); 2 Department of Polymer Engineering, Faculty of Mechanical Engineering, Budapest University of Technology and Economics, Műegyetem rkp. 3, H-1111 Budapest, Hungary; 3 FlandersMake@UGent—Core lab EEDT-DC, Technologiepark Zwijnaarde 131, B-9052 Zwijnaarde, Belgium

**Keywords:** nanoparticle-filled PTFE, thermal stability, free sintering, processing analysis

## Abstract

In this research work, unfilled and monofilled polytetrafluoroethylene (PTFE) were investigated. The applied fillers were graphene, alumina (Al_2_O_3_), boehmite alumina (BA80) and hydrotalcite (MG70). Graphene and Al_2_O_3_ are already known in the literature as potential fillers of PTFE, while BA80 and MG70 are novel fillers in PTFE. Materials were produced by room temperature pressing—free sintering method with a maximum sintering temperature of 370 °C. The mass loss and decomposition analyses were carried out by thermogravimetric analysis (TGA) in two different ways. The first was a sensitivity analysis to gain a better view into the sintering process at 370 °C maximal temperature. The second was a heating from 50 °C up to 1000 °C for a full-scale decomposition analysis. BA80 is a suitable filler for PTFE, as most of its functional groups still existed after the sintering process. Both PTFE and Al_2_O_3_ had high thermal stability. However, when Al_2_O_3_ was incorporated in PTFE, a remarkable mass loss was observed during the sintering process, which indicated that the decomposition of PTFE was catalysed by the Al_2_O_3_ filler. The observed mass loss of the Al_2_O_3_-filled PTFE was increased, as the Al_2_O_3_ content or the applied dwelling time at a 370 °C sintering temperature increased.

## 1. Introduction

Nanoparticles are widely used as reinforcement materials in thermoplastics, as they can achieve relevant improvements, e.g., mechanical, thermal and wear properties [1,2,3,4,5,6,7,8]. Polytetrafluoroethylene (PTFE), which is a semicrystalline thermoplastic, gains high importance in sliding and rolling applications, because it can work as both a matrix material and a solid lubricant [9]. PTFE has high thermal stability, excellent chemical resistance, a low coefficient of friction and good self-lubricating properties compared to other semicrystalline thermoplastics. Well-known limitations are relatively low mechanical properties and low wear resistance, which can be enhanced with the application of reinforcements such as fibres and micro- or nanoparticles [10,11,12]. Focusing on nanoparticles, graphene and alumina (Al_2_O_3_) can improve the wear resistance of PTFE by two to three orders of magnitude [13,14,15]. Due to the promising results of graphene- and Al_2_O_3_-filled PTFE, nowadays a remarkable amount of research work related to these materials is available.

Besides material properties, another relevant factor for the wear behaviour of filled PTFE is the potential chemical reactions between the given fillers, the matrix material and the metal counterfaces. In the literature, it is hypothesised that PTFE molecular chains are subjected to mechanical chain scission during the wear process, forming carboxyl functional groups (–COOH) at the end of the broken PTFE molecular chains [14,16]. For example, in case of Al_2_O_3_ fillers, these in situ formed carboxyl functional groups of PTFE can participate in complex formation with Al_2_O_3_ nanoparticles [16]. Fillers with functional groups can be promising wear resistance improvers, as they can initiate a higher number of complex formation due to their extra functional groups, which can have a positive influence on wear resistance [17]. Based on this hypothesis, boehmite alumina (BA80) and hydrotalcite (MG70) fillers are potential filler candidates, as they have high numbers of functional groups. PTFE in combination with these fillers has not yet been extensively investigated. Karger-Kocsis et al. reported in their review that boehmite does improve not only the wear resistance but also the Young’s modulus, toughness, creep resistance and thermal stability of polymer matrices [18,19].

The high processing temperature of PTFE can, however, be a challenge for those potential fillers shown above. A well-known production method for PTFE is the room temperature pressing—free sintering technique. The applied maximum sintering temperature is between 360 and 380 °C, which can be high enough to result in the decomposition of filler materials. Therefore, in case of applications of novel additives, a proper sensitivity analysis is always required. The functional groups of BA80 and MG70 fillers can be decomposed at the sintering temperature; therefore, the full sintering process is analysed with TGA to gain detailed knowledge about the thermal stability and the decomposition of the fillers at the sintering temperature.

It is reported that at a high temperature (~800 °C) the following reaction takes place between PTFE and Al_2_O_3_ (Equation (1)) [20]:

(1) $$1.5/n{\left(C_{2}F_{4}\right)}_{n}+{\mathrm{Al}}_{2}O_{3}\to 2{\mathrm{AlF}}_{3}+3\mathrm{CO}.$$

A question here is whether the range of sintering temperature can be high enough for the initiation of the mentioned reaction.

The present research work introduces the thermal analysis of graphene, Al_2_O_3_, BA80 and MG70-filled PTFE composites, focusing on the sensitivity (thermal stability) and decomposition analysis during the sintering process. In the literature, a comprehensive sensitivity analysis during the sintering process of graphene- and Al_2_O_3_-filled PTFE is rarely reported. As a result, we are lacking a general production protocol and an overview of production limitations.

## 2. Materials and Methods

### 2.1. Materials

The used PTFE powder was 3M^TM^ Dyneon^TM^ TFM^TM^ 1700 with a ~25 µm average particle size, produced by the 3M Company (Minnesota Mining and Manufacturing Company, Maplewood, MN, USA). The applied graphene was xGnP^®^ Graphene Nanoplatelets Grade M from XG Sciences (Lansing, MI, USA). The used 1015WW alpha Al_2_O_3_ with 99.5% purity was produced by Nanostructured & Amorphous Materials Inc. (Houston, TX, USA). The nanoparticle sizes were between 27 and 43 nm. The applied boehmite alumina (BA80) was Disperal^®^ 80 from Sasol (Johannesburg, South Africa) with a 35 µm average particle size and an 80 nm average crystallite size. The Al_2_O_3_ content of BA80 was 80%. Pural^®^ MG70 hydrotalcite (Mg_6_Al_2_CO_3_(OH)_16_·4(H_2_O), MG70) from Sasol (Johannesburg, South Africa) with a ~45 µm average particle size had a double-layered metal hydroxide structure including magnesium and aluminium hydroxides (70:30 wt%, respectively).

### 2.2. Production Protocol and Properties of the Unfilled and Filled PTFE Samples

The composition of the produced unfilled and filled PTFE materials can be seen in Table 1. The applied production technique was room temperature pressing—free sintering method. The PTFE and filler powders were firstly blended by intensive dry mechanical stirring, which is a less hazardous and more environment-friendly alternative than the solvent blending method. Stirring was provided by a rotating blade grinder (power: 180 W), and the stirring time was 30 s. Pressing was carried out with a Zwick Z250 universal tester at room temperature. The pressing speed was 2 mm/min, until a 12.5 MPa pressure was reached. Subsequently, 3 min of dwelling time was held at the same level of pressure. The sintering procedure was carried out in air atmosphere. The sintering cycle was shown as following: a heating rate of 90 °C/h to increase the temperature from room temperature to 370 °C, a dwelling time of 2 h at a 370 °C maximum temperature and a 30 °C/h cooling rate. All the applied fillers were incorporated with a 4, 8, 16, or 30 wt % filler content.

### 2.3. Thermal and Decomposition Analysis

#### 2.3.1. Thermogravimetric Analysis (TGA)

TGA was carried out with a TA Instruments Q500 device (New Castle, DE, USA) in nitrogen or air atmosphere, depending on the type of test. The purge gas was nitrogen with a 40 mL/min flow. The samples were placed in platinum pans and tested in a 60 mL/min nitrogen or air flow.

#### 2.3.2. Fourier-Transform Infrared Spectroscopy (FTIR)

FTIR analyses were carried out by a Bruker Tensor 37 FTIR spectrometer (Billerica, MA, USA) with a deuterated triglycine sulfate (DTGS) detector and a Specac Golden Gate single reflection monolithic diamond attenuated total reflection (ATR, Orpington, UK) sampling system. The applied spectroscopic transmission range was between 600 and 4000 cm^−1^ with a 4 cm^−1^ resolution in wavenumber.

## 3. Results and Discussion

### 3.1. Decomposition and Thermal Stability Analysis of the Applied Fillers

#### 3.1.1. Decomposition of Graphene

**Figure 1:**
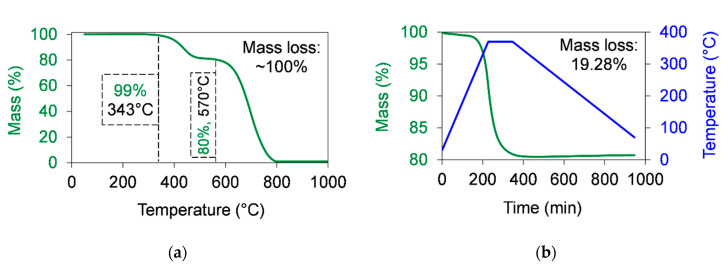
Thermal stability of graphene in air atmosphere measured by thermogravimetric analysis (TGA). (**a**) The mass loss of graphene as a function of the temperature when a 10 °C/min heating rate was used to increase the temperature up to 1000 °C. (**b**) The mass loss of graphene as a function of the time during the simulation of a sintering cycle. The sintering cycle was shown as following: A 1.5 °C/min (90 °C/h) heating rate was used to increase the temperature from room temperature up to 370 °C. Then, a dwelling time of 2 h at 370 °C was used. After that, the temperature was cooled down at a 0.5 °C/min (30 °C/h) cooling rate.

Figure 1 presents the mass loss of graphene as a function of the temperature, while Table 2 shows the residual mass (m_r_) of graphene measured by TGA. Significant decomposition was recorded only at temperatues from 343 °C, where 1% of the graphene mass was lost (Figure 1a). The decomposition procedure can be separated into two steps. The first was between ~343 and 500 °C and came from the decomposition of the amorphous carbon content [21], while the second step was in the range of ~570–800 °C and came from the decomposition of the structured graphene. The total mass loss of graphene was ~100%. The detected mass loss at the first step was ~19% (Figure 1a).

The decomposition of graphene was analysed during the sintering cycle as well. The sintering process was simulated by TGA (Figure 1b) by applying a temperature range between 30 and 370 °C at a 1.5 °C/min (90 °C/h) heating rate and a 0.5 °C/min (30 °C/h) cooling rate. The dwelling time at 370 °C was 2 h. The measured mass loss was 19.28%, which means that during the sintering cycle most of the amorphous carbon content decomposed.

#### 3.1.2. Decomposition of Al_2_O_3_

**Figure 2:**
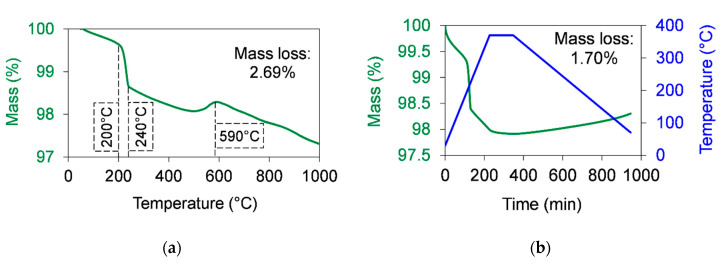
Thermal stability of alumina in air atmosphere measured by TGA. (**a**) The mass loss of graphene as a function of the temperature when a 10 °C/min heating rate was used to increase the temperature up to 1000 °C. (**b**) The mass loss of graphene as a function of the time during the simulation of a sintering cycle. The sintering cycle was shown as following: A 1.5 °C/min (90 °C/h) heating rate was used to increase the temperature from room temperature up to 370 °C. Then, a dwelling time of 2 h at 370 °C was used. After that, the temperature was cooled down at a 0.5 °C/min (30 °C/h) cooling rate.

Figure 2 and Table 2 show the mass loss of Al_2_O_3_ measured by TGA. The total mass loss was 2.69% (Figure 2a). According to the manufacturer’s datasheet, the purity of the used Al_2_O_3_ is around 99.5%. In other words, ~0.5% contaminants can be found in this filler. In the range of 200–240 °C, a higher mass loss rate was recorded, which can come from the adsorbed humidity and from some contaminants. At around 590 °C, a slight mass increase can be seen, which was supposed to come from the oxidation of some contaminants in the filler.

**Figure 3:**
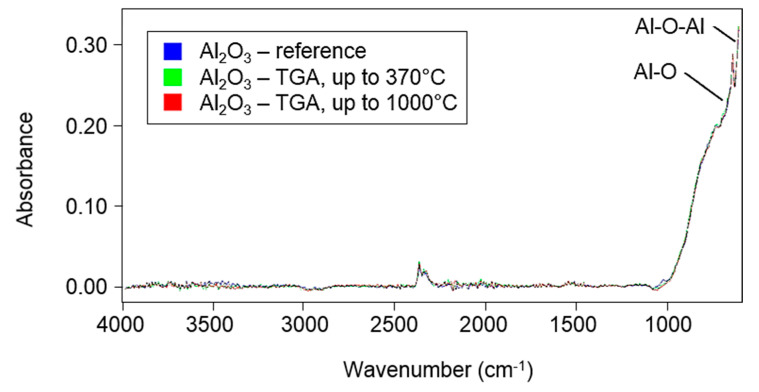
Fourier-transform infrared spectroscopy (FTIR) spectra of the reference Al_2_O_3_ (in blue), Al_2_O_3_ measured by TGA at temperatures up to 370 °C (i.e., the simulation of a sintering process) (in green) and Al_2_O_3_ measured by TGA at temperatures up to 1000 °C (in red).

The decomposition of Al_2_O_3_ was also measured during the sintering cycle. The sintering process was simulated by TGA (Figure 2b) by applying a temperature range between 30 and 370 °C at a 1.5 °C/min (90 °C/h) heating rate and a 0.5 °C/min (30 °C/h) cooling rate. The dwelling time at 370 °C was 2 h. The measured mass loss was 1.70%. The FTIR spectrum of the reference (unsintered) Al_2_O_3_ was measured. The FTIR spectra of Al_2_O_3_ at temperatures up to 370 °C (simulation of sintering process) and those of Al_2_O_3_ at temperatures up to 1000 °C were measured by TGA. No differences were found in these FTIR spectra (Figure 3).

#### 3.1.3. Decomposition of BA80 (Aluminium Hydroxide Oxide—AlO(OH))

Theoretically, BA80 decomposes into alumina and water according to the following chemical reaction (Equation (2)):

(2) $$2\mathrm{AlO}\left(\mathrm{OH}\right)\to {\mathrm{Al}}_{2}O_{3}+H_{2}O.$$

The mass percentage of $H_{2}O$ is the theoretical mass loss, which can be measured by TGA. To get information about this theoretical value, the atomic masses of aluminium (*27 Da*), oxygen (*16 Da*) and hydrogen (*1 Da*) have to been considered. In this way, the mass of the molecules in Equation (2) is introduced by Equation (3):

(3) 120 (Da)→102 (Da)+18 (Da).

The theoretical mass loss can be calculated from the mass ratio of $H_{2}O$ and 2AlO(OH) (Equation (4)):

(4) 18/120= 0.15→15%.

**Figure 4:**
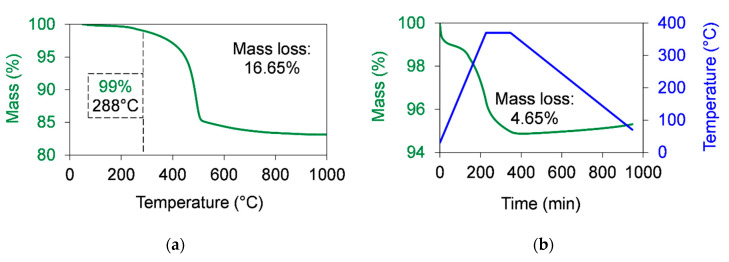
Thermal stability of boehmite alumina in air atmosphere measured by TGA. (**a**) The mass loss of graphene as a function of the temperature when a 10 °C/min heating rate was used to increase the temperature up to 1000 °C. (**b**) The mass loss of graphene as a function of the time during the simulation of a sintering cycle. The sintering cycle was shown as following: A 1.5 °C/min (90 °C/h) heating rate was used to increase the temperature from room temperature up to 370 °C. Then, a 2 h dwelling time at 370 °C was used. After that, the temperature was cooled down at a 0.5 °C/min (30 °C/h) cooling rate.

According to Equations (2)–(4), the theoretical mass percentage of water was 15%. The mass loss measured by TGA was in the range of the calculated theoretical mass loss. The measured H_2_O percentage value was 16.65% (Figure 4a and Table 2), which was slightly higher than the theoretical value. This difference comes from the humidity of and contaminants in the analysed sample. In Figure 4a, it can be seen that the decomposition of BA80 started to be relevant at temperatures from 288 °C, which was significantly lower than the applied sintering temperature (370 °C). The initiation of the decomposition was defined at a 1% mass loss, similarly to the introduced previous analyses. TGA measurement was performed with a heat hold at 200 °C to get information about the content of humidity and about these contaminants, which can vaporise under 200 °C. The total mass loss was 16.68%, which correlated well to the 16.65% mass loss (Figure 4a) presented earlier. After a 10 h dwelling time at 200 °C (10 °C/min heating rate to increase the temperature from room temperature up to 200 °C and a 10 °C/min heating rate to increase the temperature from 200 °C up to 1000 °C), the mass loss was only 0.75%, and the rest mass loss was 15.93% between 200 and 1000 °C. The percentage of the decomposed material during the sintering cycle was investigated by simulating a sintering process with TGA (Figure 4b), applying a temperature ramp between 30 and 370 °C at a 1.5 °C/min (90 °C/h) heating rate and a 0.5 °C/min (30 °C/h) cooling rate. The dwelling time at 370 °C was 2 h. The measured mass loss was 4.65%, which was around 28% (calculated by 4.65/16.65) of the total measured mass loss (Figure 4a). It means that, although some of the OH functional groups decomposed during the sintering cycle, most of them persisted. This can be beneficial in the wear process according to the introduced hypothesis, which is related to the complex formation with the carboxyl groups of PTFE chain ends during wear. To confirm the persistence of OH functional groups, the samples taken out from TGA were also analysed by FTIR.

**Figure 5:**
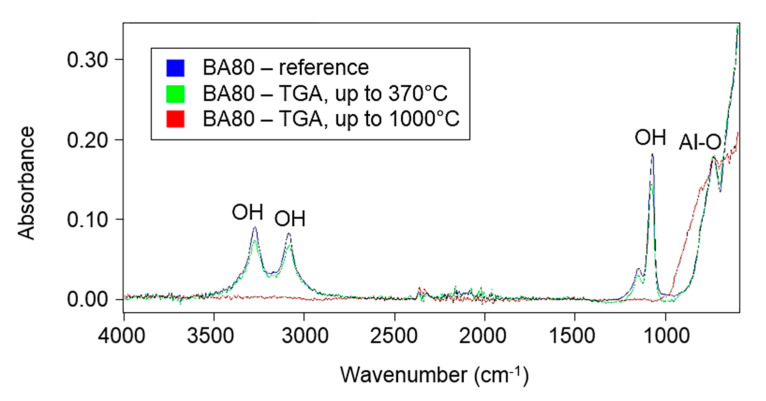
FTIR spectra of the reference BA80 (in blue), BA80 measured by TGA at temperatures up to 370 °C (i.e., simulation of a sintering process) (in green) and BA80 measured by TGA at temperatures up to 1000 °C (in red).

Figure 5 shows the FTIR spectra for the samples of the reference (unsintered) BA80, of BA80 measured by TGA at temperatures up to 370 °C (simulation of sintering process) and of BA80 measured by TGA at temperatures up to 1000 °C. The latter two samples were the same, which were analysed by TGA. Two significant peaks attributed to –OH bonds were detected in the range of 3000–3400 cm^−1^, which is in agreement with the literature [22]. These peaks can be seen in both of the reference and the sintered samples. These spectra confirmed that most of the OH functional groups survived in the sintering process, but not in the TGA test at a temperature of 1000 °C.

#### 3.1.4. Decomposition of MG70

**Figure 6:**
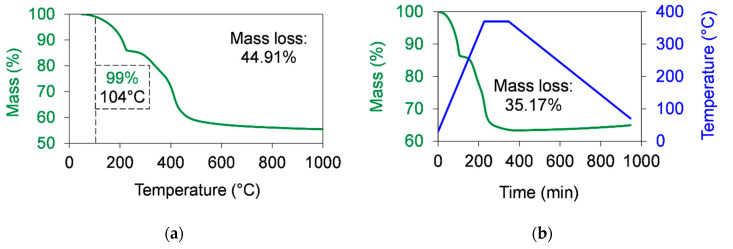
Thermal stability of MG70 measured by TGA in air atmosphere. (**a**) The mass loss of graphene as a function of the temperature when a 10 °C/min heating rate was used to increase the temperature up to 1000 °C. (**b**) The mass loss of graphene as a function of the time during the simulation of a sintering cycle. The sintering cycle was shown as following: A 1.5 °C/min (90 °C/h) heating rate was used to increase the temperature from room temperature up to 370 °C. Then, a 2 h dwelling time at 370 °C was used. After that, the temperature was cooled down at a 0.5 °C/min (30 °C/h) cooling rate.

Figure 6 and Table 2 shows the mass percentages and the residual mass (m_r_) of MG70 measured by TGA. Significant decomposition was recorded at temperatures from 104 °C, where 1% of the graphene mass was lost (Figure 6a). The total mass loss during the MG70 decomposition was 44.91% (Figure 6a), while this value in case of the simulated sintering procedure was 35.17% (Figure 6b). It means that the mass loss during the sintering process was around ~78% of the full mass loss of MG70. Due to the observed high decomposition of MG70 during the sintering process, this material is not a promising filler for high temperature production of PTFE.

**Figure 7:**
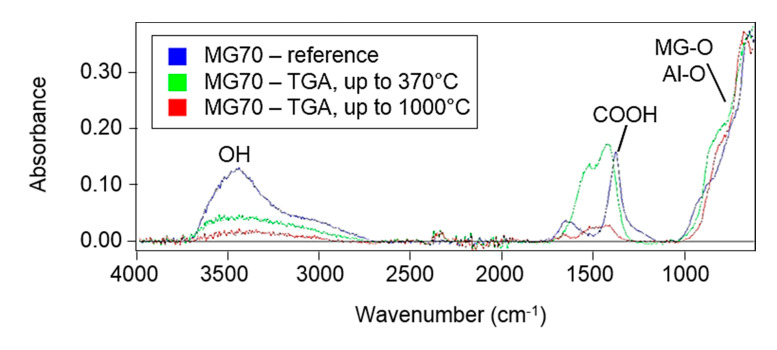
FTIR spectra of the reference MG70 (in blue), MG70 measured by TGA at temperatures up to 370 °C (i.e., simulation of the sintering process) (in green) and MG70 measured by TGA at temperatures up to 1000 °C (in red).

Figure 7 compares the FTIR spectrum of the reference (unsintered) MG70, that of MG70 measured by TGA at temperatures up to 370 °C (i.e., simulation of a sintering process) and that of MG70 measured by TGA at temperatures up to 1000 °C. The peaks of the sintered MG70 already changed significantly compared to those of the reference material.

### 3.2. Decomposition and Thermal Stability Analysis of the Developed Unfilled/Filled Materials

#### 3.2.1. Decomposition of the Unfilled and Filled PTFE during the Full Sintering Process

Table 2 and Table 3 show the residual mass (m_r_) in case of the neat fillers and the filled PTFE. The sintering process involved a 90 °C/h heating rate to increase the temperature up to 370 °C, a 2 h dwelling time at a 370 °C temperature and a 30 °C/h cooling rate to decrease the temperature to room temperature. The residual mass was evaluated at the beginning of the hold time, at the beginning of the cooling (after 2 h heat dwelling) and after the final sintering process. As shown in Table 3, the theoretical sample mass was calculated from the measured residual mass of the neat PTFE and the neat fillers. The highlighted numbers in Table 3 (Section 4) reflected these final m_r_ values, where the difference between theoretical and measured values was higher than 1%.

As can be seen, there was a gap between the theoretical and the measured residual masses of the developed composites. In case of graphene- and Al_2_O_3_-filled samples, the measured residual mass was higher than it was expected from the mass losses of the PTFE and the given fillers. The most significant difference was observed in case of the PTFE/Al_2_O_3_-30 sample. These higher values indicated that there was an interaction between the graphene or Al_2_O_3_ filler and PTFE, and consequently, a higher decomposition was observed compared to those of the neat additives.

#### 3.2.2. Extended Heat Dwelling (10 h)

Decomposition analysis of PTFE composites was performed by simulating a sintering process with a 10 h dwelling time at a maximum temperature of 370 °C. The heating and cooling rates were the same as in the original sintering process. The residual masses was observed in Table 4 at the start of the dwelling time (0 h) and after 2, 4, 6, 8 and 10 h dwelling times. With this long interval, it was possible to get a more detailed insight into the stability of the composites during the sintering process. As can be seen in Table 4 and Table 5, the masses of graphene-, Al_2_O_3_- and BA80-filled samples were significantly decreased with the increasing dwelling time during the full heating dwelling time of 10 h. In contrast with this, only slight mass losses were observed in case of the unfilled PTFE and the neat Al_2_O_3_, which were only 0.07% and 0.13% during the 10 h dwelling time at a 370 °C temperature, respectively. The PTFE/MG70 samples showed moderate mass decreases in the 10 h interval, because most of the decomposition occurred at the heating period before the dwelling time. The most significant influence of the dwelling time for the decomposition was observed in case of the PTFE/Al_2_O_3_-30 sample, where the mass loss was 16.60%. The theoretical value based on the neat materials and the filler contents was only 0.09% (Table 5), which means that the rest of the material loss came from an interaction between PTFE and Al_2_O_3_. These results clearly showed that, in case of the applied fillers, a longer dwelling time led to a higher decomposed material mass, which can have a negative effect on the final material properties. The largest difference between the measured and theoretical values was observed for the Al_2_O_3_-filled samples in case of 4, 8, 16 and 30 wt % filler contents as well (highlighted numbers in Table 5).

Regarding the potential interaction between PTFE and Al_2_O_3_, the FTIR spectra of the sintered PTFE/Al_2_O_3_-30 sample after a 10 h heat dwelling time did not indicate the existence of AlF_3_ bonds (Equation (1)). Therefore, the introduced reaction was supposed not to take place at the sintering circumstances. A potential explanation for the measured high mass loss of the Al_2_O_3_-filled samples can be a catalysing effect of the Al_2_O_3_ filler on the decomposition of the PTFE material.

### 3.3. Decomposition and Thermal Stability Analysis of the Developed Unfilled and Filled Materials

**Figure 8:**
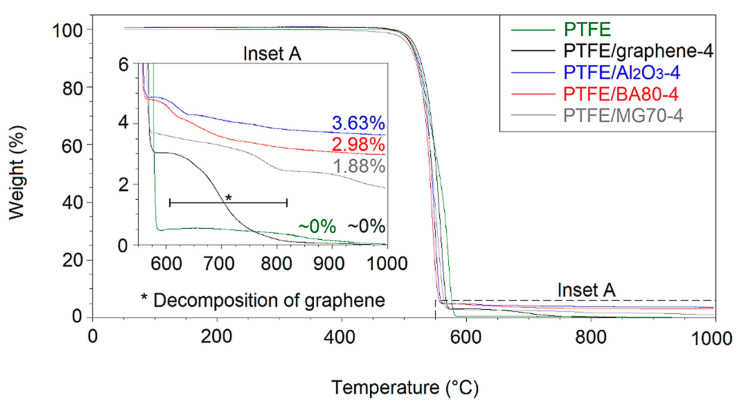
Decomposition analysis of the filled and unfilled PTFE in air atmosphere measured by TGA.

**Figure 9:**
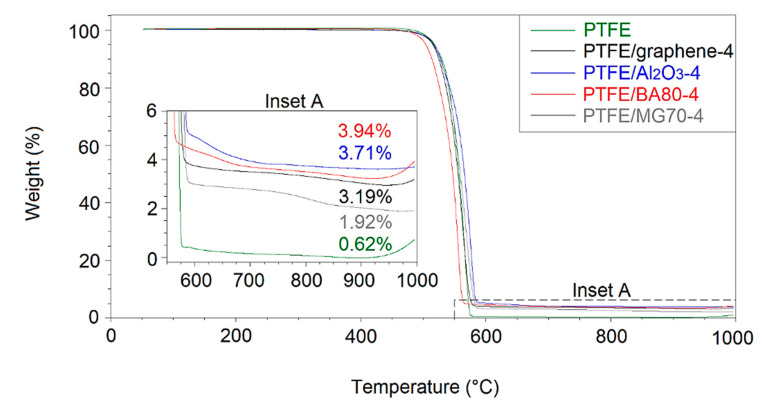
Decomposition analysis of the filled and unfilled PTFE in nitrogen atmosphere measured by TGA.

The decomposition analyses of the developed unfilled and filled PTFE in air and nitrogen atmosphere are shown in Figure 8 and Figure 9, respectively. All the filled materials included fillers in a nominal content of 4 wt %. The residual mass percentages in air atmosphere correlated well with the TGA measurements of the neat additives (Figure 1a, Figure 2a, Figure 4a and Figure 6a). In case of the neat PTFE and PTFE/graphene-4, the observed decomposition was 100%. In PTFE/graphene-4, an approximately 3% mass loss was observed in the range of 600–800 °C, which correlated well with the second step of the graphene decomposition (Figure 1a).

Table 6 and Table 7 show the temperatures measured at 1%, 10% and 50% mass losses for all analysed materials in air and nitrogen atmosphere, respectively. The temperature of all the additives decreased at 1% and 10% mass losses with respect to that of the reference neat PTFE, except the PTFE/graphene-4 sample, where the temperature at a 10% mass loss was higher than in case of the neat PTFE. These results indicated lower thermal stability in both air and nitrogen atmosphere.

## 4. Conclusions

In this research, the thermal and decomposition analyses of neat PTFE, graphene-, Al_2_O_3_-, BA80- and MG70-filled PTFE were conducted by using the high temperature production method.

The decompositions of the neat PTFE and the neat fillers during a sintering process were measured by TGA (maximum sintering temperature: 370 °C) followed by FTIR analysis:

- As it was expected, the neat PTFE and the neat Al_2_O_3_ filler had high thermal stability during the sintering process.
- Most of the hydroxyl (OH) functional groups of the BA80 filler survived in the applied sintering process.
- The thermal stability of MG70 was too low to be used in the sintered PTFE.

The decompositions of the filled PTFE materials during a sintering process were measured by TGA (maximum sintering temperature: 370 °C) followed by FTIR analysis:

- Low thermal stability was observed in case of the Al_2_O_3_-filled PTFE materials; the mass losses of these materials were remarkably higher than was expected from the results of the neat PTFE and Al_2_O_3_. This indicated that there was an interaction between the thermally stable PTFE and Al_2_O_3_ at 370 °C. The decomposition extent of these materials was increased, as the filler content increased.
- The sintering process with a 10 h dwelling time confirmed the low thermal stability of the Al_2_O_3_-filled PTFE materials. These materials were sensitive to the applied dwelling time at the maximum sintering temperature, unlike the neat PTFE or neat Al_2_O_3_. The FTIR spectra did not confirm the existence of AlF_3_ (Equation (1)). Therefore, it was supposed that the Al_2_O_3_ filler catalysed the decomposition of the PTFE matrix.

## Figures and Tables

**Table 1:** The developed neat polytetrafluoroethylene (PTFE) and PTFE-based materials.

Materials	Matrix	Filler	Filler Content (wt %)
PTFE	PTFE	-	-
PTFE/graphene-4	PTFE	graphene	4
PTFE/graphene-8	PTFE	graphene	8
PTFE/graphene-16	PTFE	graphene	16
PTFE/graphene-30	PTFE	graphene	30
PTFE/Al_2_O_3_-4	PTFE	alumina (Al_2_O_3_)	4
PTFE/Al_2_O_3_-8	PTFE	alumina (Al_2_O_3_)	8
PTFE/Al_2_O_3_-16	PTFE	alumina (Al_2_O_3_)	16
PTFE/Al_2_O_3_-30	PTFE	alumina (Al_2_O_3_)	30
PTFE/BA80-4	PTFE	boehmite alumina (BA80)	4
PTFE/BA80-8	PTFE	boehmite alumina (BA80)	8
PTFE/BA80-16	PTFE	boehmite alumina (BA80)	16
PTFE/BA80-30	PTFE	boehmite alumina (BA80)	30
PTFE/MG70-4	PTFE	hydrotalcite (MG70)	4
PTFE/MG70-8	PTFE	hydrotalcite (MG70)	8
PTFE/MG70-16	PTFE	hydrotalcite (MG70)	16
PTFE/MG70-30	PTFE	hydrotalcite (MG70)	30

**Table 2:** Residual mass (m_r_) of the filled PTFE after the sintering process and at 1000 °C measured by TGA.

Materials	m_r_ after Sintering (%)	m_r_ at 1000 °C (%)
PTFE	99.99	~0
graphene	80.72	~0
Al_2_O_3_	98.30	97.31
BA80	95.35	83.35
MG70	64.83	55.09

**Table 3:** Residual mass (m_r_) during the sintering process measured by TGA.

Materials	m_r_ at the Beginning of Hold Time at 370 °C (%)	m_r_ at the Beginning of Cooling at 370 °C (%)	Final m_r_ after Sintering (%)
Section 1: Experimental values based on the neat PTFE and the neat fillers measured by TGA			
PTFE	100.00	99.97	99.99
graphene	91.35	80.93	80.72
Al_2_O_3_	98.03	97.96	98.30
BA80	96.37	95.01	95.35
MG70	71.26	63.42	64.83
Section 2: Experimental values based on the filled PTFE measured by TGA			
PTFE/graphene-30	97.40	92.63	92.15
PTFE/graphene-16	98.65	95.92	95.60
PTFE/graphene-8	99.04	97.63	97.62
PTFE/graphene-4	99.59	98.86	98.86
PTFE/Al_2_O_3_-30	99.22	96.28	95.35
PTFE/Al_2_O_3_-16	99.83	98.64	98.20
PTFE/Al_2_O_3_-8	99.73	98.92	98.66
PTFE/Al_2_O_3_-4	99.64	99.25	99.17
PTFE/BA80-30	99.07	97.96	97.91
PTFE/BA80-16	99.39	98.85	98.66
PTFE/BA80-8	99.65	99.34	99.32
PTFE/BA80-4	99.75	99.48	99.45
PTFE/MG70-30	91.51	89.58	89.54
PTFE/MG70-16	95.19	93.93	94.11
PTFE/MG70-8	97.71	97.10	96.97
PTFE/MG70-4	98.58	98.23	98.32
Section 3: Theoretical values calculated based on the filler contents and m_r_ in Section 1			
PTFE/graphene-30	97.41	94.26	94.21
PTFE/graphene-16	98.62	96.92	96.91
PTFE/graphene-8	99.31	98.45	98.45
PTFE/graphene-4	99.65	99.21	99.22
PTFE/Al_2_O_3_-30	99.41	99.37	99.48
PTFE/Al_2_O_3_-16	99.68	99.65	99.72
PTFE/Al_2_O_3_-8	99.84	99.81	99.85
PTFE/Al_2_O_3_-4	99.92	99.89	99.92
PTFE/BA80-30	98.91	98.48	98.60
PTFE/BA80-16	99.42	99.18	99.25
PTFE/BA80-8	99.71	99.57	99.62
PTFE/BA80-4	99.85	99.77	99.80
PTFE/MG70-30	91.38	89.01	89.44
PTFE/MG70-16	95.40	94.12	94.36
PTFE/MG70-8	97.70	97.05	97.18
PTFE/MG70-4	98.85	98.51	98.58
Section 4: Difference between the theoretical and measured values (= Section 3 − Section 2)			
PTFE/graphene-30	0.01	1.63	2.06 *
PTFE/graphene-16	−0.03	1.00	1.31 *
PTFE/graphene-8	0.27	0.82	0.83
PTFE/graphene-4	0.06	0.35	0.36
PTFE/Al_2_O_3_-30	0.19	3.09	4.13 *
PTFE/Al_2_O_3_-16	−0.15	1.01	1.52 *
PTFE/Al_2_O_3_-8	0.11	0.89	1.19 *
PTFE/Al_2_O_3_-4	0.28	0.64	0.75
PTFE/BA80-30	−0.16	0.52	0.69
PTFE/BA80-16	0.03	0.33	0.59
PTFE/BA80-8	0.06	0.23	0.30
PTFE/BA80-4	0.10	0.29	0.35
PTFE/MG70-30	−0.13	−0.58	−0.10
PTFE/MG70-16	0.21	0.19	0.25
PTFE/MG70-8	−0.01	−0.05	0.21
PTFE/MG70-4	0.27	0.28	0.26

**Table 4:** Residual masses during different dwelling times at 370 °C in air atmosphere measured by TGA.

Materials	Residual Mass (m_r_) at an Elapsed Dwelling Time (%)					
	0 h	2 h	4 h	6 h	8 h	10 h
PTFE	99.96	99.92	99.91	99.90	99.90	99.89
graphene	90.80	80.37	79.57	79.33	79.19	79.09
Al_2_O_3_	97.92	97.81	97.79	97.79	97.79	97.79
BA80	96.65	95.32	94.83	94.46	94.14	93.84
MG70	69.18	64.17	63.44	62.99	62.80	62.61
PTFE/graphene-30	97.17	92.33	90.97	90.27	89.70	89.19
PTFE/Al_2_O_3_-30	99.30	96.37	92.85	89.40	86.00	82.70
PTFE/BA80-30	98.83	97.38	95.93	94.74	93.68	92.73
PTFE/MG70-30	90.85	88.90	88.47	88.15	87.89	87.66
PTFE/graphene-16	98.39	95.65	94.93	94.46	94.04	93.64
PTFE/Al_2_O_3_-16	99.65	97.84	96.04	94.38	92.84	91.38
PTFE/BA80-16	99.40	98.65	97.75	96.85	95.98	95.14
PTFE/MG70-16	95.27	94.16	93.91	93.72	93.56	93.43
PTFE/graphene-8	99.19	97.79	97.25	97.10	96.90	96.71
PTFE/Al_2_O_3_-8	99.73	98.79	97.77	96.86	95.97	95.12
PTFE/BA80-8	99.71	99.26	98.69	98.10	97.49	96.89
PTFE/MG70-8	97.54	96.93	96.79	96.70	96.62	96.56
PTFE/graphene-4	99.61	99.02	98.83	98.65	98.47	98.26
PTFE/Al_2_O_3_-4	99.87	99.44	99.02	98.58	98.16	97.73
PTFE/BA80-4	99.76	99.31	98.86	98.41	98.00	97.61
PTFE/MG70-4	98.73	98.41	98.33	98.27	98.21	98.16

**Table 5:** Mass losses during a dwelling time of 10 h at 370 °C in air atmosphere measured by TGA. The theoretical values in column 3 were calculated based on the basis of filler contents and the mass loss of the neat PTFE and the neat fillers. Column 4 introduces the difference between the theoretical (column 3) and measured values (column 2).

Materials	Mass Loss (0–10 h) Measured by TGA (%)	Theoretical Values of Mass Loss (0–10 h) (%)	Mass Loss (0–10 h) Difference (%) (Measured Value—Theoretical Value)
PTFE	0.07	---	---
graphene	11.71	---	---
Al_2_O_3_	0.13	---	---
BA80	2.81	---	---
MG70	6.57	---	---
PTFE/graphene-30	7.98	3.56	4.42
PTFE/Al_2_O_3_-30	16.60	0.09	16.51 *
PTFE/BA80-30	6.10	0.89	5.21
PTFE/MG70-30	3.19	2.02	1.17
PTFE/graphene-16	4.75	1.93	2.82
PTFE/Al_2_O_3_-16	8.27	0.08	8.19 *
PTFE/BA80-16	4.26	0.51	3.75
PTFE/MG70-16	1.84	1.11	0.73
PTFE/graphene-8	2.48	1.00	1.48
PTFE/Al_2_O_3_-8	4.61	0.07	4.54 *
PTFE/BA80-8	2.82	0.29	2.53
PTFE/MG70-8	0.98	0.59	0.39
PTFE/graphene-4	1.35	0.54	0.81
PTFE/Al_2_O_3_-4	2.14	0.07	2.07 *
PTFE/BA80-4	2.15	0.18	1.97
PTFE/MG70-4	0.57	0.33	0.24

**Table 6:** The temperatures in case of 1%, 10% and 50% mass losses in air atmosphere measured by TGA.

Materials	Temperature at a 1% Mass Loss (°C)	Temperature at a 10% Mass Loss (°C)	Temperature at a 50% Mass Loss (°C)
PTFE	489.9	521.2	556.4
PTFE/graphene-4	488.4	524.3	551.7
PTFE/Al_2_O_3_-4	485.2	518.7	544.1
PTFE/BA80-4	483.9	518.1	540.9
PTFE/MG70-4	464.3	516.2	545.3

**Table 7:** The temperatures in case of 1%, 10% and 50% mass losses in nitrogen atmosphere measured by TGA.

Materials	Temperature at a 1% Mass Loss (°C)	Temperature at a 10% Mass Loss (°C)	Temperature at a 50% Mass Loss (°C)
PTFE	492.8	528.2	556.7
PTFE/graphene-4	488.4	524.4	555.5
PTFE/Al_2_O_3_-4	482.7	526.3	565.6
PTFE/BA80-4	479.5	513.0	546.6
PTFE/MG70-4	484.6	527.6	558.6

## References

[1] Song P., Wang C., Chen L., Zheng Y., Liu L., Wu Q., Huang G., Yu Y., Wang H. (2017). Thermally stable, conductive and flame-retardant nylon 612 composites created by adding two-dimensional alumina platelets. Compos. Part A.

[2] Liang J.Z. (2019). Effects of tension rates and filler size on tensile properties of polypropylene/ graphene nano-platelets composites. Compos. Part B.

[3] Rodriguez V., Sukumaran J., Schlarb A.K., De Baets P. (2016). Reciprocating sliding wear behaviour of PEEK-based hybrid composites. Wear.

[4] Gumede T.P., Luyt A.S., Müller A.J. (2018). Review on PCL, PBS, and PCL/PBS blends containing carbon nanotubes. Express Polym. Lett..

[5] Jun Y.S., Um J.G., Jiang G., Yu A. (2018). A study on the effects of graphene nano-platelets (GnPs) sheet sizes from a few to hundred microns on the thermal, mechanical, and electrical properties of polypropylene (PP)/GnPs composites. Express Polym. Lett..

[6] Pan C., Kou K., Zhang Y., Li Z., Wu G. (2018). Enhanced through-plane thermal conductivity of PTFE composites with hybrid fillers of hexagonal boron nitride platelets and aluminum nitride particles. Compos. Part B.

[7] Pan C., Zhang J., Kou K., Zhang Y., Wu G. (2018). Investigation of the through-plane thermal conductivity of polymer composites with in-plane oriented hexagonal boron nitride. Int. J. Heat. Mass. Tran..

[8] Cui J., Zhou Z., Jia M., Chen X., Shi C., Zhao N., Guo X. (2020). Solid Polymer Electrolytes with Flexible Framework of SiO2 Nanofibers for Highly Safe Solid Lithium Batteries. Polymers.

[9] Kalácska G. (2013). An engineering approach to dry friction behaviour of numerous engineering plastics with respect to the mechanical properties. Express Polym. Lett..

[10] Aderikha V.N., Krasnov A.P., Shapovalov V.A., Golub A.S. (2014). Peculiarities of tribological behavior of low-filled composites based on polytetrafluoroethylene (PTFE) and molybdenum disulphide. Wear.

[11] Makowiec M.E., Blanchet T.A. (2017). Improved wear resistance of nanotube- and other carbon-filled PTFE composites. Wear.

[12] Khedkar J., Negulescu I., Meletis E.I. (2002). Sliding wear behavior of PTFE composites. Wear.

[13] Krick B.A., Ewin J.J., Blackman G.S., Junk C.P., Sawyer W.G. (2012). Environmental dependence of ultra-low wear behavior of polytetrafluoroethylene (PTFE) and alumina composites suggests tribochemical mechanisms. Tribol. Int..

[14] Padenko E., van Rooyen L.J., Wetzel B., Karger-Kocsis J. (2016). “Ultralow” sliding wear polytetrafluoro ethylene nanocomposites with functionalized graphene. J. Reinf. Plast. Compos..

[15] Kandanur S.S., Rafiee M.A., Yavari F., Schrameyer M., Yu Z.Z., Blanchet T.A., Koratkar N. (2012). Suppression of wear in graphene polymer composites. Carbon.

[16] Harris K.L., Pitenis A.A., Sawyer W.G., Krick B.A., Blackman G.S., Kasprzak D.J., Junk C.P. (2015). PTFE tribology and the role of mechanochemistry in the development of protective surface films. Macromolecules.

[17] Padenko E., van Rooyen L.J., Karger-Kocsis J. (2017). Transfer film formation in PTFE/oxyfluorinated graphene nanocomposites during dry sliding. Tribol. Lett..

[18] Karger-Kocsis J., Lendvai L. (2017). Polymer/boehmite nanocomposites: A review. J. Appl. Polym. Sci..

[19] Pedrazzoli D., Khumalo V.M., Karger-Kocsis J., Pegoretti A. (2014). Thermal, viscoelastic and mechanical behavior of polypropylene with synthetic boehmite alumina nanoparticles. Polym. Test..

[20] Hobosyan M.A., Kirakosyan K.G., Kharatyan S.L., Martirosyan K.S. (2015). PTFE-Al_2_O_3_ reactive interaction at high heating rates. J. Therm. Anal. Calorim..

[21] Chen S., Xin Y., Zhou Y., Zhang F., Ma Y., Zhou H., Qi L. (2014). Branched CNT @ SnO_2_ nanorods @ carbon hierarchical heterostructures for lithium ion batteries with high reversibility and rate capability. J. Mater. Chem. A.

[22] Liu C., Shih K., Gao Y., Li F., Wei L. (2012). Dechlorinating transformation of propachlor through nucleophilic substitution by dithionite on the surface of alumina. J. Soils. Sediments.

